# Polychromatic Supplemental Lighting from underneath Canopy Is More Effective to Enhance Tomato Plant Development by Improving Leaf Photosynthesis and Stomatal Regulation

**DOI:** 10.3389/fpls.2016.01832

**Published:** 2016-12-09

**Authors:** Yu Song, Chengyao Jiang, Lihong Gao

**Affiliations:** ^1^College of Horticulture, China Agricultural UniversityBeijing, China; ^2^Institute of Germplasm Resources, Xinjiang Academy of Agricultural ScienceUrumqi, China; ^3^Graduate School of Horticulture, Chiba UniversityMatsudo, Japan

**Keywords:** light insufficiency, supplemental lighting, composite spectrums, underneath and inner canopy, leaf photosynthesis, stomatal regulation, plant growth

## Abstract

Light insufficient stress caused by canopy interception and mutual shading is a major factor limiting plant growth and development in intensive crop cultivation. Supplemental lighting can be used to give light to the lower canopy leaves and is considered to be an effective method to cope with low irradiation stress. Leaf photosynthesis, stomatal regulation, and plant growth and development of young tomato plants were examined to estimate the effects of supplemental lighting with various composite spectra and different light orientations. Light-emitting diodes (LEDs) of polychromatic light quality, red + blue (R/B), white + red + blue (W/R/B), white + red + far-red (W/R/FR), and white + blue (W/B) were assembled from the underneath canopy or from the inner canopy as supplemental lighting resources. The results showed that the use of supplemental lighting significantly increased the photosynthetic efficiency, and reduced stomatal closure while promoting plant growth. Among all supplemental lighting treatments, the W/R/B and W/B from the underneath canopy had best performance. The different photosynthetic performances among the supplemental lighting treatments are resulted from variations in CO_2_ utilization. The enhanced blue light fraction in the W/R/B and W/B could better stimulate stomatal opening and promote photosynthetic electron transport activity, thus better improving photosynthetic rate. Compared with the inner canopy treatment, the supplemental lighting from the underneath canopy could better enhance the carbon dioxide assimilation efficiency and excessive energy dissipation, leading to an improved photosynthetic performance. Stomatal morphology was highly correlated to leaf photosynthesis and plant development, and should thus be an important determinant for the photosynthesis and the growth of greenhouse tomatoes.

## Introduction

Although controlled by genetic characteristics, the morphology, and development of plants are sensitive to fluctuations in environmental factors. Light is one of the most important variables regulating biological reactions and material accumulation in plants (Kopsell and Kopsell, 2008). Currently, intensive cultivation schedules have been adopted in greenhouse crop production to achieve high yield. However, this can result in insufficient light reaching the lower canopies and altered plant morphogenesis and photosynthesis (Hogewoning et al., 2010a; Terfa et al., 2013). In greenhouse tomato cultivation, the light interception of each canopy layer decreases sharply down the plant profile, and mutual shading also occurs (Acock et al., 1978; Xu et al., 1997; Lu et al., 2012a; Tewolde et al., 2016). No more than 35% of the total intercepted solar light reaches the leaves under the tomato fruit trusses (Cockshull et al., 1992; Lu et al., 2012a), and such a shortage of light triggers an extremely low net photosynthetic rate and premature leaf senescence (Acock et al., 1978; Xu et al., 1997).

Supplemental lighting, using artificial light resources and employed in lower canopies, is considered to be an efficient method for relieving low-light stress on plants. Numerous studies the effects of application of supplemental lighting have been conducted on various species via aspects of the canopy layer (Hovi et al., 2004; Hovi and Tahvonen, 2008; Pettersen et al., 2010), light source (Lu et al., 2012a,b; Piringer and Cathey, 1960), light intensity (Demers et al., 1998; Dorais, 2003), and light period (Piringer and Cathey, 1960; Tewolde et al., 2016). Among those, the selection of optimized light wavelength is more complex and is often reported with mixed results (Okamoto et al., 1996; Li and Kubota, 2009; Ni et al., 2009; Lu et al., 2012b). For example, blue light suppresses hypocotyl elongation in wheat (Goins et al., 1997) and tomato (Massa et al., 2008), but improves the dry matter production and the photosynthetic capacity in pepper (Brown et al., 1995), wheat (Goins et al., 1997), and spinach (Matsuda et al., 2007). In contrast, red light seems to be most effective in the biomass assimilation of lettuce (Yanagi et al., 1996; Kim et al., 2006), but not spinach and radish (Okamoto et al., 1996; Yorio et al., 2001). Similarly, different red/far-red ratios show contrary results in phytochemical content (Alokam et al., 2002) and plant photomorphogenesis (Kirdmanee et al., 1993; Brown et al., 1995; Runkle and Heins, 2001). These results have shown the viability of optimizing the light quality in promoting plant morphology and productivity to eventually improve the greenhouse economic benefits. However, previous studies have mainly examined only a few selected sole light qualities or the compound spectrum with only the combination of red/blue or red/far-red at one time, and there are no reports examining the effects of polychromatic light quality (the combination of white, blue, red, and far-red) affecting plant growth and development. Therefore, it is necessary to investigate the polychromatic light quality effects when provided as supplemental lighting resource applied for horticultural crop production.

Photosynthesis is sensitive to light condition and essential for plant growth. Improved leaf photosynthesis would enhance plant development (Hovi et al., 2004; Hovi and Tahvonen, 2008; Pettersen et al., 2010). The investigation of leaf structure–function relationships in photosynthesis shows that internal maximum photosynthesis rates were not close to the leaf surface of the upper epidermis, where light intensity was highest, but instead occurred in the middle and lower palisade layers (Nishio et al., 1993; Evans, 1995; Sun et al., 1998; Sun and Nishio, 2001; Evans and Vogelmann, 2003). These deeper layers have higher electron transport activities and greater amounts of photosynthetic proteins (Terashima and Inoue, 1985; Terashima and Evans, 1988; Sun and Nishio, 2001). This indicated that the supplemental lighting from the underneath canopy (with light orientation to the abaxial epidermis) might function better in improving leaf and plant development than using the supplemental lighting from the inner canopy (with light orientation to the adaxial epidermis).

Stomatal regulation, which is highly correlated with leaf photosynthesis, governs the overall CO_2_ assimilation, and water loss from plants (Casson and Hetherington, 2010; Araújo et al., 2011). Stomatal behavior can be affected by the light wavelength through energy conversion (Shimazaki et al., 2007; Chen et al., 2012), membrane ion transport (Fan et al., 2004; Araújo et al., 2011), and metabolic activity in guard cells (Talbott and Zeiger, 1993; Mott et al., 2008; O'Carrigan et al., 2014). Tomato (*Lycopersicon esculentum* Mill.) is regarded as one of the most important horticultural crops in the world and previous researches have demonstrated the distinct influence of supplemental lighting quality on plant development (Demers et al., 1998; Massa et al., 2008), which consequently affects fruit developing speed (Lu et al., 2012b), yield (Lu et al., 2012b; Gómez et al., 2013), and quality (Dorais, 2003; Lu et al., 2012b; Olle and Viršile, 2013), and eventually determined the economic applicability of the application of this technique. In addition, the stomata are mostly distributed on the abaxial epidermis of tomato leaves. We thus hypothesized that supplemental lighting from the underneath canopy have more obvious improvement on the stomatal regulation and photosynthesis capacity, which might consequently better stimulate plant growth and development, compared with supplemental lighting from the inner canopy. White light has a higher penetration rate through the tomato canopy than other colors (Lu et al., 2012b), blue light could contribute a larger stomata size (Sharkey and Raschke, 1981; Loreto et al., 2009) in leaves and a higher health index of tomato plants (Chang et al., 2010; Chen et al., 2014), and red/blue and red/far-red light are the most commonly used in horticulture cultivation. Thus, we added white light to the spectrum in this study to investigate the effects of polychromatic supplemental lighting. Plants undergo the most vegetative growth before anthesis and the fluctuation of environment factors would cause largely morphological changes at this stage (Poorter and Garnier, 1996; Matsuda et al., 2014). Therefore, in this study, to understand how plants respond to the interaction of light quality and light orientation, we treated young tomato plants with/without supplemental lighting with different polychromatic light quality levels orientated from underneath or inner canopy for 4 weeks, and investigated the resulting fluctuations in leaf photosynthesis and stomatal behavior, as well as the consequent response of plant morphologic development.

## Materials and methods

### Plant material and growth conditions

The experiment was conducted in a glass greenhouse (Venlo-type, with double spans and a north–south orientation) in Urumqi, China (43°46′12″N, 87°40′48″E) from September, 2015 to April, 2016. Tomato (“NS3389,” Agricultural Science and Technology Co. Ltd., Guangzhou, China) seeds were sown into trays with commercial substrate (Peilei No. 2, Peile Organic Fertilizer Co., Zhenjiang, China) and germinated in an environmentally controlled box (RTOP-1000D, Top Yun Co. Ltd., Hanzhou, China) for 24 days. Other environment factors were fixed, including the photosynthetic photon flux density (PPFD), photocycle, day/night temperatures, and CO_2_ concentration of 350 μmol·m^−2^·s^−1^, 16 h, 23/18°C, and 800 μmol·mol^−1^, according to Matsuda et al. (2011, 2014). The trays were sub-irrigated every other day, with a commercial nutrient solution at an electrical conductivity of 1.5 dS·m^−1^.

At 24 days after sowing, each seedling was transplanted into the greenhouse at a plant density of 16.6 plant·m^−2^ with an automatically irrigated nutrient solution (Nakano et al., 2010). The greenhouse environment was maintained, with daytime mean air temperature of 27 ± 2°C, a night-time mean air temperature of 20 ± 2°C, and a daily mean relative humidity above 60%. Although the CO_2_ concentration was not measured, it was assumed to be close to the outside level, based on measurements in the same season in another year (data not shown).

### Supplemental lighting treatment

**Figure 1:**
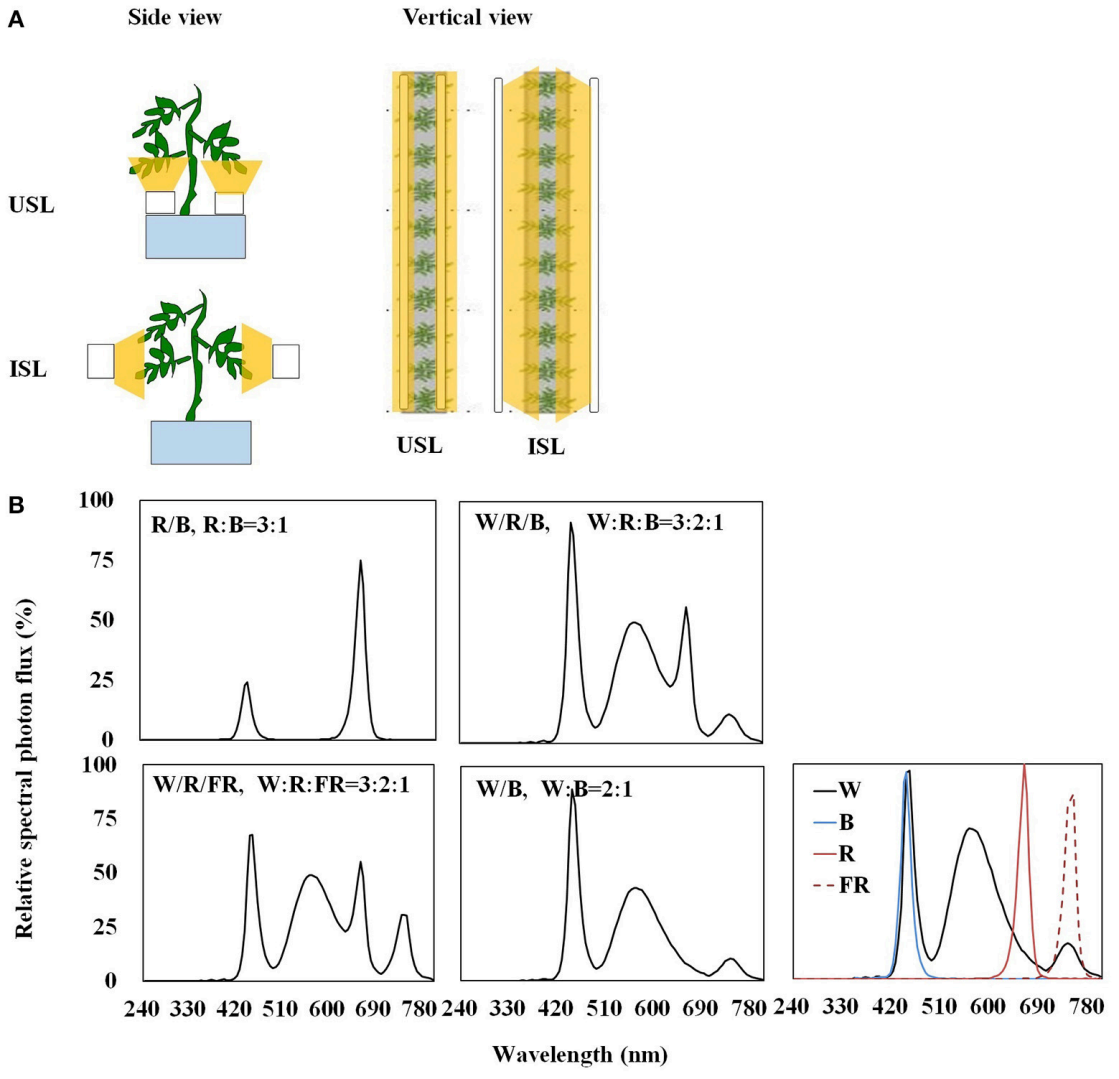
**Schematic diagram (A)** and relative spectral photon flux of polychromatic LEDs **(B)** of the supplemental lighting treatment in this experiment. Supplemental lighting from the underneath canopy (USL) or from the inner canopy (ISL) was applied to plants from the time of transplantation while a no supplemental lighting condition was considered to be the control. Each supplemental lighting module was kept fixed at a 10 cm distance to the abaxial or adaxial epidermis of the leaf, respectively, with a PPFD of 200 μmol·m^−2^ ·s^−1^. The light quality contains red + blue (R/B, R:B = 3:1), white + red + blue (W/R/B, W: R:B = 3:2:1), white + red + far-red (W/R/FR, W:R:FR = 3:2:1) and white + blue (W/B, W:B = 2:1). The spectral property of each LED module used for polychromatic LEDs combination also shown in **(B)**. The wavelengths of the light sources were recorded at 240–800 nm with a spectrometer (SR9910-v7, Irradiant Ltd., Tranent, UK). A digital timer, dimmer, and transformer were used to maintain the light period (16 h, GMT +8 8:00–24:00) and light intensity.

Light-emitting diodes (LEDs; Philips Co. Ltd., Netherlands) were used as the light source. Four polychromatic light, red + blue (R/B, R:B = 3:1), white + red + blue (W/R/B, W: R:B = 3:2:1), white + red + far-red (W/R/FR, W:R:FR = 3:2:1), and white + blue (W/B, W:B = 2:1), were applied from two light orientations: supplemental lighting from the underneath and inner canopy (Figure 1). LEDs were fixed to movable girders that ensured the lighting distance from the adaxial epidermis of inner canopy leaves or the abaxial epidermis of the lowest leaf truss was maintained at 10 cm. The measured PPFD was 200 μmol·m^−2^·s^−1^ at 10 cm from the LED module. Plants without supplemental lighting were considered to be the control plants. Each treatment consisted of three bench rows of plants, with each row containing 20 plants, with a 16 h photo cycle each day (during 8:00–24:00 at GMT +8, which is 6:00–22:00 local time).

### Gas-exchange parameter measurements

Gas-exchange measurements were conducted on the second terminal leaflets of leaves on the fifth youngest node (Matsuda et al., 2014) with a portable photosynthesis system (Li-6400XT; Li-Cor Inc., Lincoln, NE, USA) during 11:00–16:00, GMT +8 (9:00–14:00, local time) on the 28^th^ day after transplanting. The net photosynthetic rate (*P*_N_), stomatal conductance (*G*_s_), transpiration rate (*T*_r_), and intercellular CO_2_ concentration (*C*_i_) were measured. Measurements were conducted with PPFD, leaf temperature, CO_2_ concentration, and relative humidity at 800 ± 5 μmol·m^−2^·s^−1^, 28 ± 1°C, 400 ± 2 μmol·m^−2^, and 63 ± 2%, respectively.

The light and CO_2_ response curve measurement was conducted to calculate the light-saturated maximum photosynthetic rate (*P*_Nmax_), apparent quantum yield (AQY), CO_2_-saturated maximum photosynthetic rate (A_max_), and carboxylation efficiency (CE). The leaf temperature was set at 25°C, and the PPFD and CO_2_ concentrations ranged from 1600 to 0 μmol·m^−2^·s^−1^and 1200 to 0 μmol·mol^−1^, respectively. The *P*_N_–PPFD and *P*_N_–*C*_*i*_ curves were plotted using a non-linear curving-fitting routine with the *P*_N_ data and the corresponding light intensity or intercellular CO_2_ concentration, respectively. Indexes were identified as *P*_Nmax_ and *A*_max_, the maximum net photosynthetic rate at the saturation light intensity and CO_2_ concentration, respectively (Bassman and Zwier, 1991). AQY were the initial slope of the *P*_*N*_−PPFD (Lambers et al., 2008; Skillman, 2008), and CE also known as *V*_*cmax*_, the maximum velocity of ribulose 1,5-bisphosphate carboxylase/oxygenase (Rubisco) for carboxylation, which can be calculated from *P*_N_−*C*_i_ curve curves according to equation from FvCB model (Farquhar et al., 1980; Sharkey et al., 2007):

(1) $$\begin{array}{l} P_{N}=V_{cmax}\left[\frac{C_{c}-\Gamma^{*}}{C_{c}+K_{c}\left(1+O/K_{o}\right)}\right]-R_{d} \end{array}$$

where *V*_*cmax*_ is the maximum velocity of Rubisco for carboxylation, *C*_*c*_ is the CO_2_ partial pressure at Rubisco, *K*_*c*_ is the Michaelis constant of Rubisco for carbon dioxide, O is the partial pressure of oxygen at Rubisco, and *K*_*o*_ is the inhibition constant (usually taken to be the Michaelis constant) of Rubisco for oxygen. The symbol Γ^*^ is the photorespiratory compensation point and *R*_*d*_ is day respiration. This equation lends itself to a linear regression approach to estimating *V*_*cmax*_ as the slope and −*R*_*d*_ as the intercept.

### Chlorophyll fluorescence parameter measurements

Chlorophyll fluorescence parameters were measured to evaluate the light absorption, electron transfer, thermal dissipation, and excitation distribution in the photosystem of tomato plants treated with or without supplemental lighting from the underneath or inner canopy with polychromatic LEDs after the adaption of the leaves to stable light or dark states. Leaf chlorophyll fluorescences levels were measured simultaneously using a portable photosynthesis system (Li-6400XT, Li-Cor Inc.) with an integrated fluorescence fluorometer (Li 6400-40 leaf chamber fluorometer, Li-Cor Inc.). The gas supply (ambient CO_2_ concentrations and 21% O_2_), actinic light (LED with 90% red light, 630 nm; 10% blue light, 470 nm) and measurement light (630 nm, 1 μmol·m^−2^·s^−1^) were all setting in accordance with Sun et al. (2016). The steady state chlorophyll fluorescence level (*F*_s_), minimum chlorophyll fluorescence at the open PSII center (*F*_o_, dark treated; *F*′_o_, light adapted), maximum chlorophyll fluorescence at the closed PSII center (*F*_m_, dark treated; *F*′_m_, light adapted) were all determined in accordance with the work of Kramer et al. (2004). The maximum quantum yield of the PSII primary photochemistry [*F*_v_/*F*_m_; (*F*_m_−*F*_o_)/*F*_m_], efficiency of excitation energy capture by open PSII reaction centers [*F*′_v_/*F*′_m_ = (*F*′_m_ − *F*′_o_)/*F*′_m_], quantum yield of the PSII electron transport [ΦPSII; (*F*′_m_ − *F*_s_)/ = *F*′_m_], and non-photochemical quenching [NPQ = (*F*_m_ − *F*′_m_)/ *F*′_m_] were calculated from the measured parameters (Maxwell and Johnson, 2000).

### Stomatal assays

Stomatal assays were carried out essentially as described in work of O'Carrigan et al. (2014) and conducted on abaxial epidermal strips of the leaves at the same position of photosynthesis measurement on the 29^th^ day after transplanting during 11:00–16:00, GMT +8 (9:00–14:00, local time). The samples were peeled, immersed in a transparent nail polish buffer, and mounted on glass slides before micro-imaging. Images of each epidermal strip were taken under a Leica microscope (Leica Microsystems AG, Solms, Germany) fitted with a Nikon NIS-F1 CCD camera and a Nikon DS-U3 controller (Nikon, Tokyo, Japan), and analyzed with a Nikon NIS Element software. Stomatal density was defined as the number of stomata per mm^2^ and stomatal index was calculated as ([number of stomata]/[number of epidermal cells + number of stomata]) × 100 (Kubínová, 1994). The stomatal aperture width and length was defined in **Figure 5**, and stomatal pore area was calculated by assuming an oval pore shape according to Chen et al. (2010, 2012).

### Plant growth analyses

On the 30^th^ day after transplantation, plants were destructively harvested for the determination of the dry weights of the shoots and roots, the height and diameter of stems, health index, leaf areas, specific leaf area (leaf area per unit leaf mass), leaf chlorophyll contents, flower number, and carbohydrate determination. Plants were washed with distilled water and weighed after wiping the water off. The leaf area per plant was measured using a leaf area meter (LI-3000C; Li-Cor Inc.). The leaf chlorophyll content was determined using a chlorophyll meter (SPAD-502; Minolta, Osaka, Japan). Samples were oven dried at 80°C until a constant weight was attained, and the dry weight subsequently recorded. The health index, widely used as general evaluation of the young plant growth quality (Fujii, 1952, 1953; Lu et al., 1984; Ge, 1987; Zhang et al., 1992; Song, 1999; Yang and Zhou, 2010; Huang et al., 2012; Chen et al., 2014), was calculated as (stem diameter/stem height) × total dry weight, according to Fan et al. (2013). The carbohydrates, including the soluble-sugar and starch, were measured in samples of the milled leaf material. Soluble sugars were extracted with 80% (v/v) ethanol at 80°C and their contents were determined enzymatically, and starch in the 80% ethanol-insoluble fraction was extracted and digested, and the resultant glucose content was assayed by Nelson–Somogyi's method (Matsuda et al., 2014).

### Statistical analyses

Duncan's multiple range test was performed at *P* < 0.05 for among all treatments and two-way analysis of variance (ANOVA) was performed at *P* < 0.05 with supplemental light quality and light orientation as sources of variation. SPSS 11.0 software (SPSS Inc., Chicago, IL, USA) was used for the statistical analyses.

## Results

### Gas-exchange parameter

**Figure 2:**
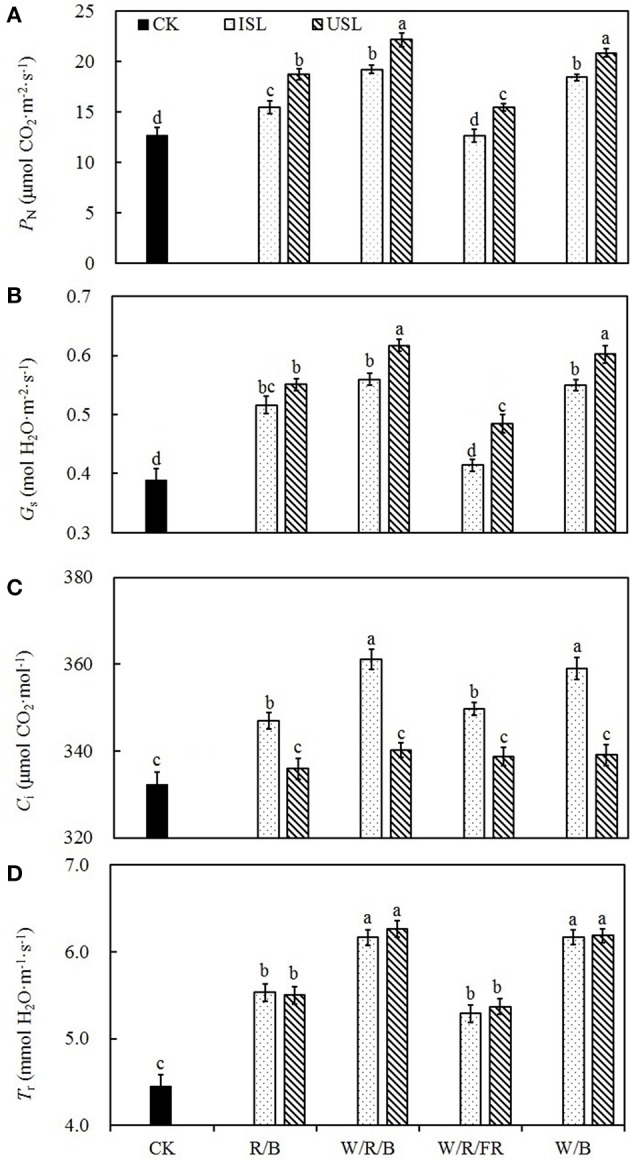
**The effects of different treatments on the net photosynthetic rate (*P*_N_; A)**, stomatal conductance (*G*_s_; B), intercellular CO_2_ concentration (*C*_i_; C), and transpiration rate (*T*_r_; D) in the leaves of tomato plants. Supplemental lighting from the underneath canopy (USL) or from the inner canopy (ISL) was applied to plants from the time of transplantation while a no supplemental lighting condition was considered to be the control (CK). Parameters were measured on the second terminal leaflets of leaves on the fifth youngest node for each treatment. Measurements were conducted with PPFD, leaf temperature, CO_2_ concentration, and relative humidity at 800 ± 5 μmol·m^−2^·s^−1^, 28 ± 1°C, 400 ± 2 μmol·m^−2^, and 63 ± 2%, respectively. Means ± SE (*n* = 8) different letters indicate significant differences at *P* < 0.05 according to Duncan's multiple range test.

**Figure 3:**
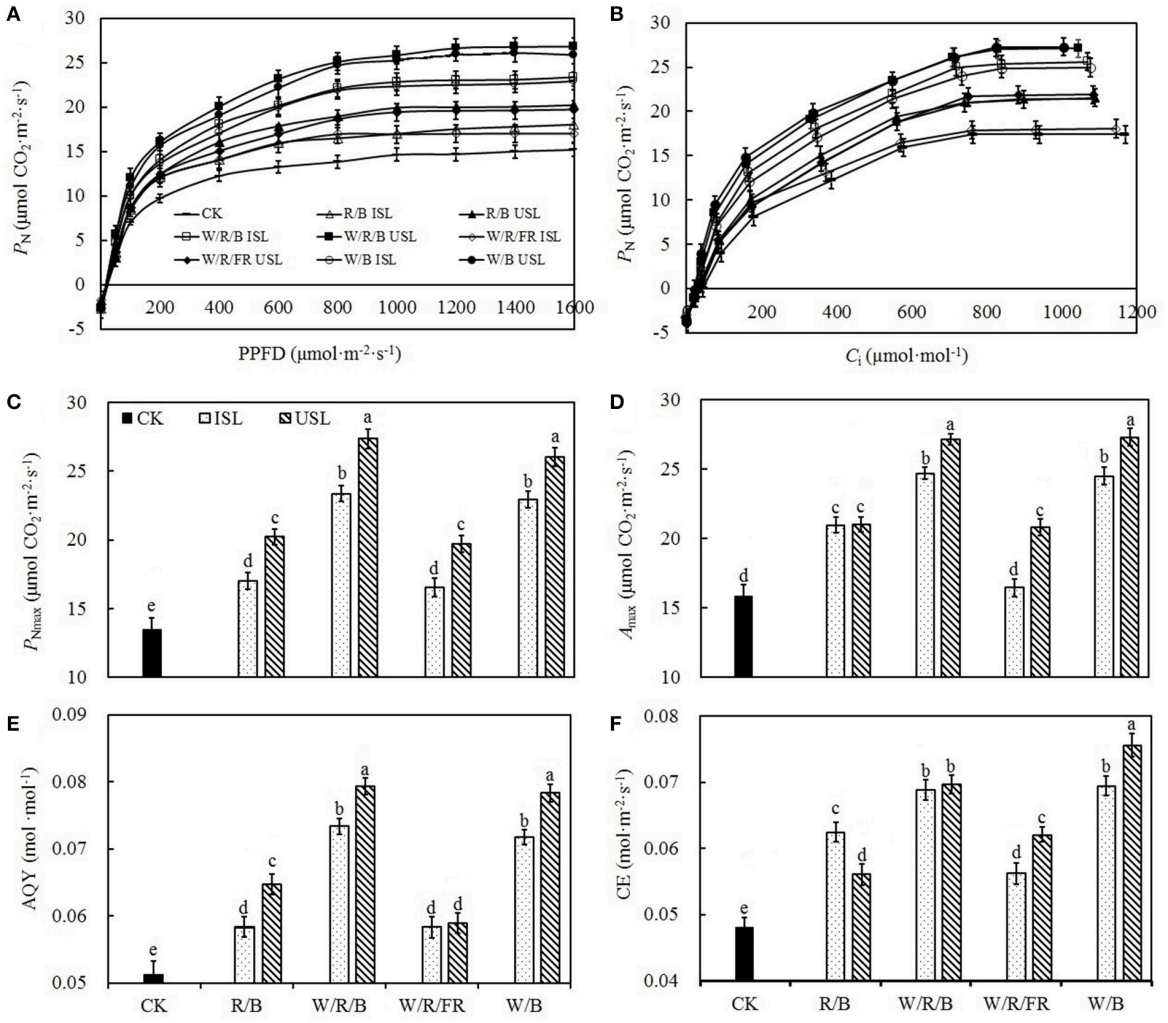
**The effects of different treatments on the light response curve (A)**, CO_2_ response curve **(B)**, light-saturated maximum photosynthetic rate (*P*_Nmax_; **C**), CO_2_-saturated maximum photosynthetic rate (*A*_max_; **D**), apparent quantum yield (AQY; **E**), and carboxylic efficiency (CE; **F**) in the leaves of tomato plants. Supplemental lighting from the underneath canopy (USL) or from the inner canopy (ISL) was applied to plants from the time of transplantation while a no supplemental lighting condition was considered to be the control (CK). Parameters were measured on the second terminal leaflets of leaves from the fifth youngest node for each treatment. Means ± *SE* (*n* = 8) different letters indicate significant differences at *P* < 0.05 according to Duncan's multiple range test.

Compared with the control, regardless of the light quality, the supplemental lighting from the inner canopy significantly increased the *P*_N_, *G*_s_, *C*_i_, and *T*_r_ (Figures 2A–D). In contrast, while the supplemental lighting from the underneath canopy significantly increased the *P*_N_, *G*_s_, and *T*_r_, it had no significant effects on *C*_i_. Among the supplemental lighting treatments, as regard of light quality, data in the W/R/B and W/B was significantly higher than those in the R/B and W/R/FR. The leaf photosynthesis capacity was significantly promoted by supplemental lighting. Compared with the control, supplemental lighting significantly increased the *P*_Nmax_, *A*_max_, AQY, and CE (Figure 3). Among the supplemental lighting treatments, the data in the supplemental lighting from the underneath canopy were generally higher than those in the supplemental lighting from the inner canopy and data in the W/R/B and W/B were significantly higher than those in the R/B and W/R/FR. The *P*_Nmax_, *A*_max_, and AQY were highest in the W/R/B from treatments of supplemental lighting from the underneath canopy and increased by 86.5, 70.0, 53.6%, respectively, compared with the control (Figures 3C–E). The CE was highest in the W/B from treatment of supplemental lighting from the underneath canopy and increased 57.5% compared with the control (Figure 3F).

### Chlorophyll fluorescence parameter

**Figure 4:**
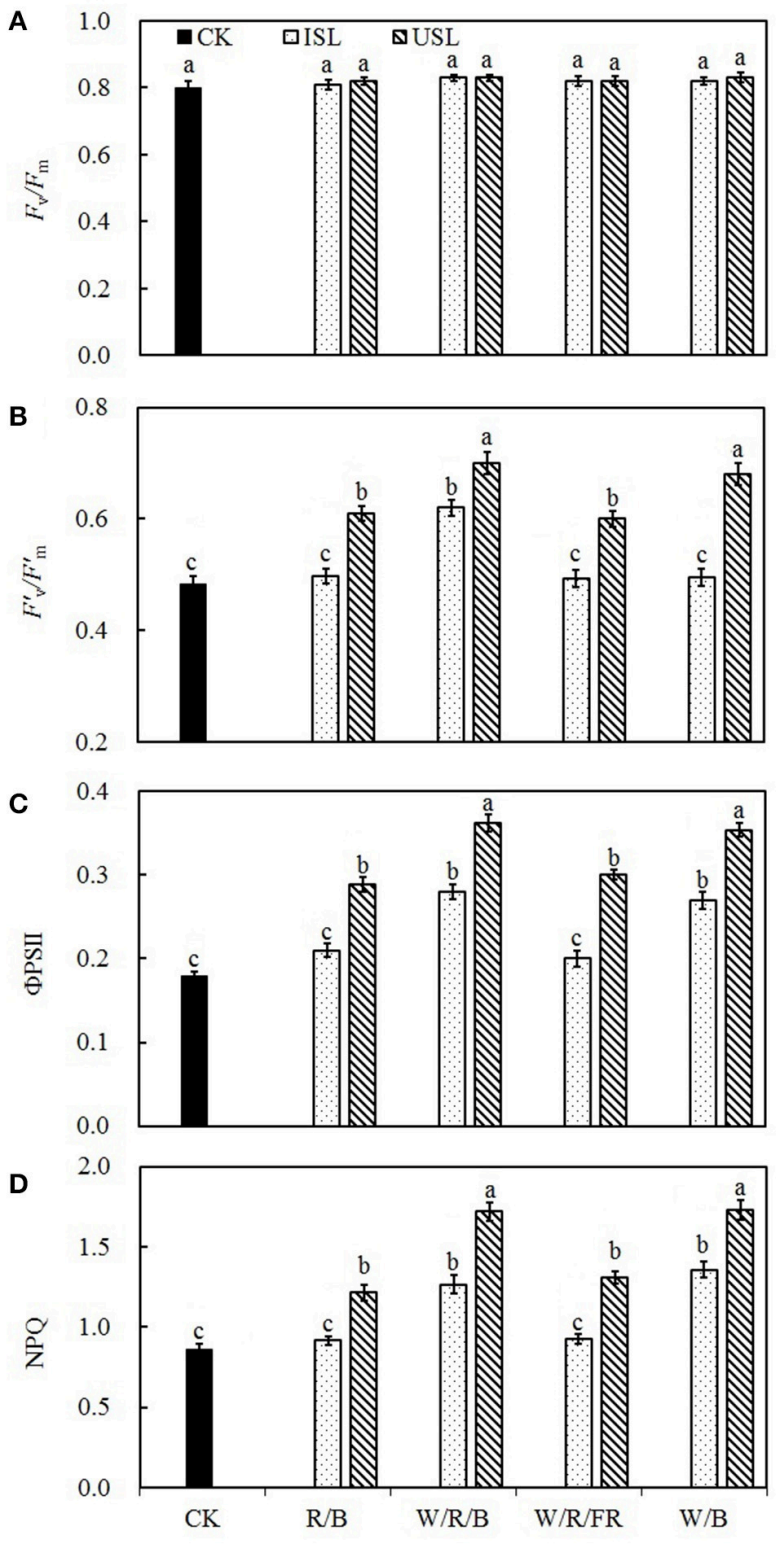
**The effects of different treatments on maximum quantum yield of the PSII primary photochemistry (*F*_v_/*F*_m_; A)**, the efficiency of excitation energy capture by PSII (*F*′_v_/*F*′_m_; **B**), the quantum yield of PSII electron transport (ΦPSII; **C**), and non-photochemical quenching (NPQ; **D**) in leaves of tomato plants. Supplemental lighting from the underneath canopy (USL) or from the inner canopy (ISL) was applied to plants from the time of transplantation while a no supplemental lighting condition was considered to be the control (CK). Parameters were measured on the second terminal leaflets of leaves on the fifth youngest node for each treatment. Means ± *SE* (*n* = 8) different letters indicate significant differences at *P* < 0.05 according to Duncan's multiple range test.

Compared with the control, the supplemental lighting from the underneath canopy significantly increased the efficiency of the excitation energy captured by the open PSII reaction centers (*F*′_v_/*F*′_m_; Figure 4B), the quantum yield of the PSII electron transport (ΦPSII; Figure 4C), and the non-photochemical quenching (NPQ; Figure 4D), but had no effect on the maximum quantum yield of the PSII primary photochemistry (*F*_v_/*F*_m_; Figure 4A). Under treatment of supplemental lighting from the inner canopy, the W/R/B and W/B significantly increased ΦPSII and NPQ but had no effect on *F*_v_/*F*_m_, *F*′_v_/*F*′_m_, while R/B and W/R/FR had no effect on any of the indexes.

### Stomatal characteristics

**Figure 5:**
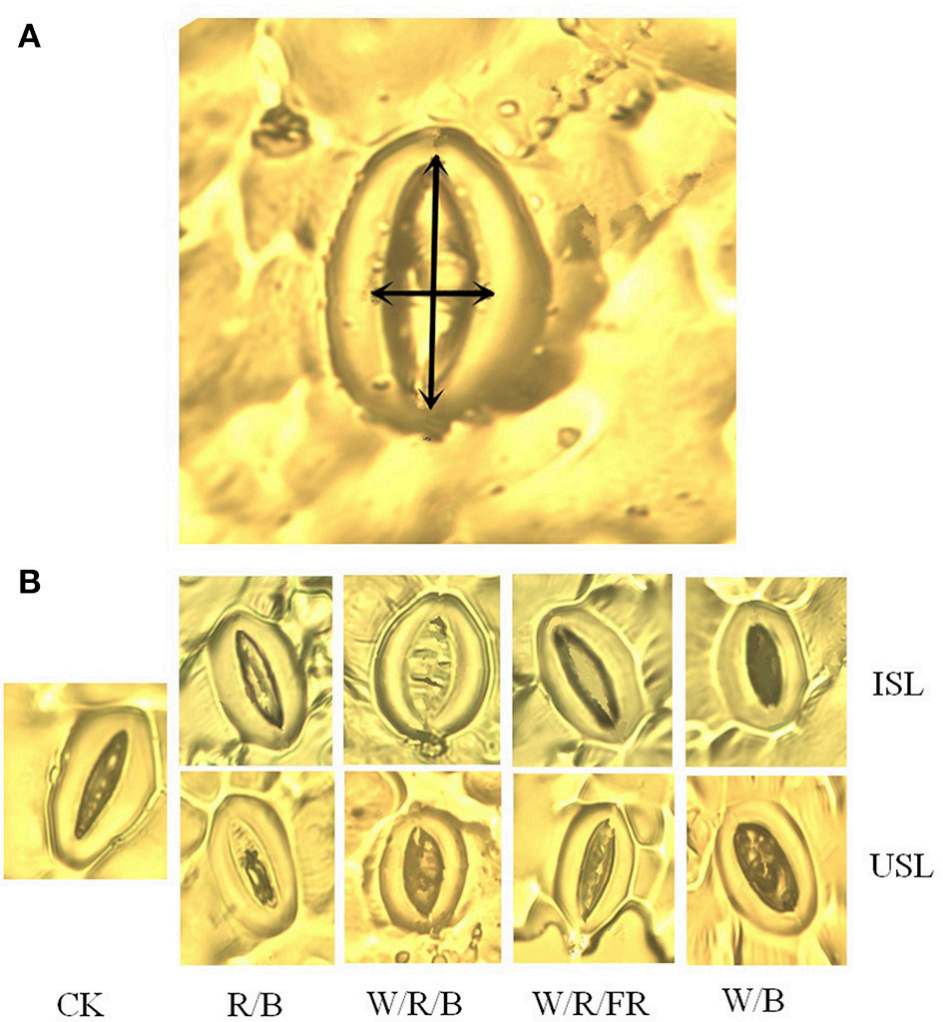
**Representative stomatal images for the measurements of stomatal parameters**. Supplemental lighting from the underneath canopy (USL) or from the inner canopy (ISL) was applied to plants from the time of transplantation while a no supplemental lighting condition was considered to be the control (CK). **(A)** A light micrograph of stomata aperture width (horizontal arrow) and aperture length (vertical arrow) are indicated by double arrows. **(B)** Typical stomata aperture closure responses to different treatments in this experiment are shown.

**Figure 6:**
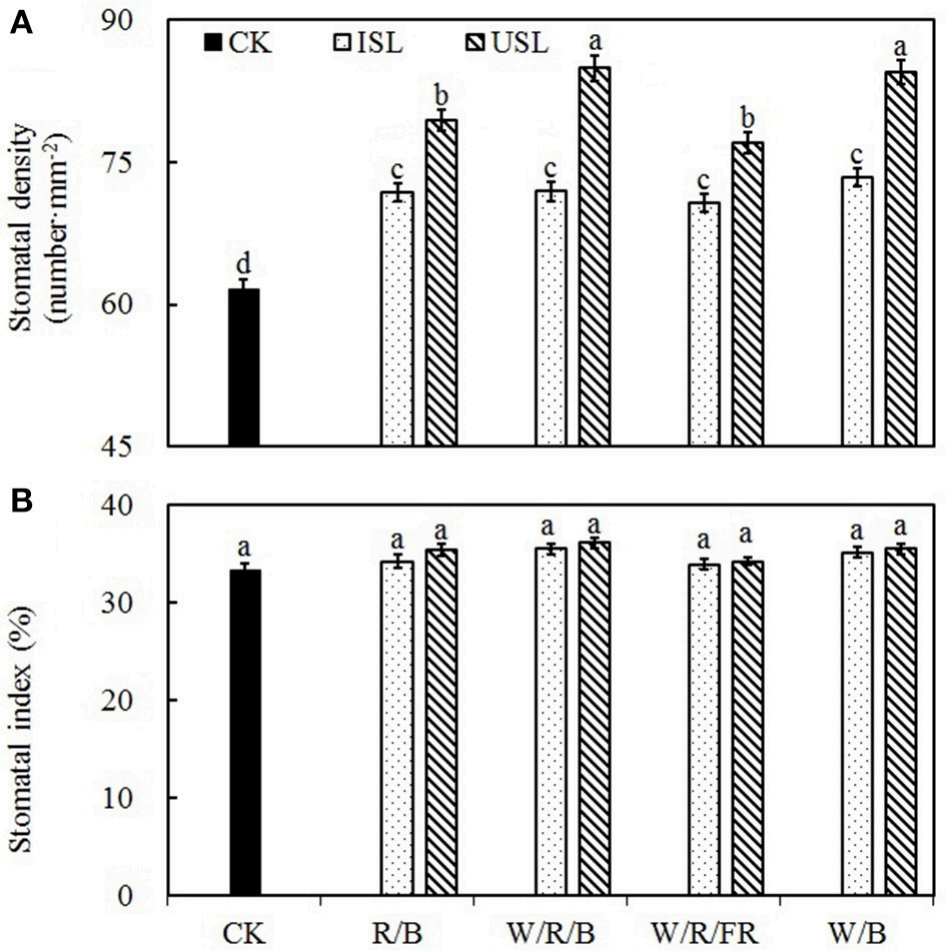
**The effects of different treatments on the stomatal density (A)** and stomatal index **(B)** in the leaves of tomato plants. Supplemental lighting from the underneath canopy (USL) or from the inner canopy (ISL) was applied to plants from the time of transplantation while a no supplemental lighting condition was considered to be the control (CK). Parameters were measured on the second terminal leaflets of leaves on the fifth youngest node for each treatment. Means ± *SE* (*n* = 16) different letters indicate significant differences at *P* < 0.05 according to Duncan's multiple range test.

**Figure 7:**
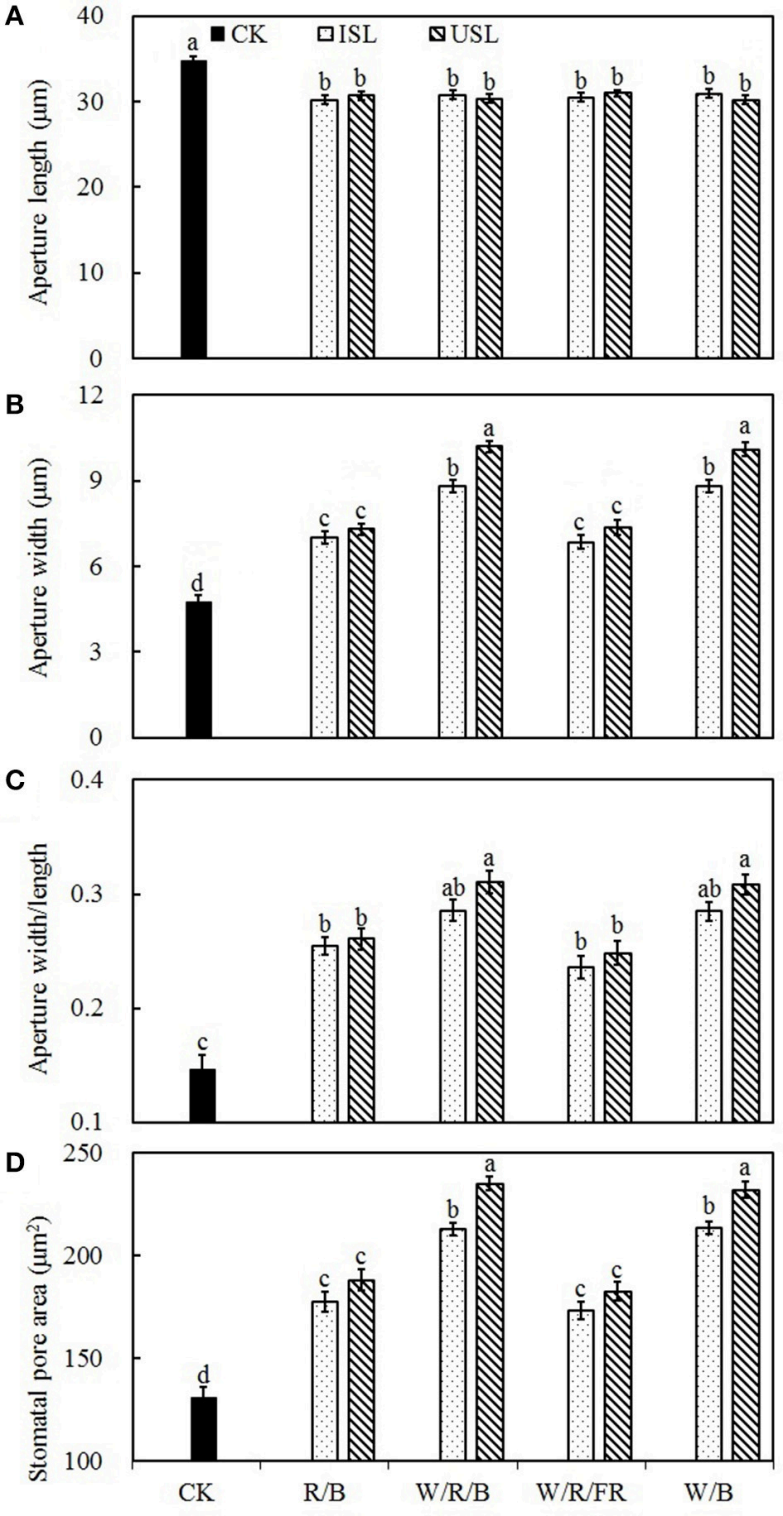
**The effects of different treatments on the aperture length (A)**, aperture width **(B)**, aperture width/length **(C)**, and stomatal pore area **(D)** in the leaves of tomato plants. Supplemental lighting from the underneath canopy (USL) or from the inner canopy (ISL) was applied to plants from the time of transplantation while a no supplemental lighting condition was considered to be the control (CK). Parameters were measured on the second terminal leaflets of leaves on the fifth youngest node for each treatment. Means ± *SE* (*n* = 16) different letters indicate significant differences at *P* < 0.05 according to Duncan's multiple range test.

Compared with the control, the supplemental lighting significantly increased stomatal density but not influenced stomatal index (**Figure 6**). The data on the stomatal density in the treatments of supplemental lighting from the underneath canopy were significantly higher than those in the treatments of supplemental lighting from the inner canopy, while no significant difference among the light quality was observed. The stomatal aperture size was significantly affected by the supplemental lighting (Figures 5, **7**). The aperture length was significantly decreased, while the aperture width was significantly increased when the leaf was exposed to supplemental lighting (**Figures 7A,B**), resulting in a significantly higher width/length ratio and a larger stomatal pore area (**Figures 7C,D**). The data in the W/R/B and W/B were generally higher than those in the R/B and W/R/FR, and the data in the treatments of supplemental lighting from the underneath canopy were generally higher than those in the treatments of supplemental lighting from the inner canopy.

### Plant growth and carbohydrate accumulation

Supplemental lighting had positive effects on the plant growth and carbohydrate accumulation (Table 1). With the exception of the stem height, the data in supplemental lighting were obviously higher than the data in the control. Generally, the data in the W/R/B and W/B were higher than those in other light quality and the data in the treatments of supplemental lighting from the underneath canopy were higher than the inner canopy treatments. With the excepted of the leaf area, the light quality, light orientation, or quality × orientation had a significant influence on the measured index.

**Table 1:** **Effects of polychromatic supplemental lighting quality and light orientation on tomato plant morphological characteristics and carbohydrate accumulation**.

**Treatment**		**Shoot dry weight (g)**	**Root dry weight (g)**	**Stem height (cm)**	**Stem diameter (mm)**	**Leaf area (m^2^)**	**Specific leaf area (cm^2^·g^−1^)**	**Health index**	**SPAD**	**Flower number**	**Soluble sugar content (mg.g^−1^)**	**Starch content (mg.g^−1^)**
**Light quality**	**Light orientation**											
CK		19.03 ± 0.86e	2.06 ± 0.06e	85.55 ± 0.77a	13.0 ± 0.34e	0.22 ± 0.04a	104.65 ± 1.04e	0.32 ± 0.03f	40.03 ± 1.21b	1.1 ± 0.11d	78.3 ± 9.41d	35.3 ± 1.41d
R/B	ISL	25.32 ± 0.43c	2.43 ± 0.03d	80.63 ± 0.67b	13.2 ± 0.12de	0.23 ± 0.03a	120.21 ± 0.81c	0.45 ± 0.02de	45.23 ± 0.64a	1.7 ± 0.13b	126.3 ± 4.54c	43.6 ± 0.91c
	USL	29.01 ± 0.51b	3.18 ± 0.04b	72.25 ± 0.41de	14.3 ± 0.22bc	0.24 ± 0.03a	120.28 ± 0.62c	0.63 ± 0.02b	45.14 ± 0.61a	1.8 ± 0.22b	125.7 ± 4.61c	44.1 ± 0.64c
W/R/B	ISL	28.97 ± 0.54b	3.15 ± 0.03b	76.88 ± 0.25cd	14.0 ± 0.14c	0.23 ± 0.04a	127.98 ± 0.44b	0.58 ± 0.02bc	45.55 ± 0.33a	2.3 ± 0.23a	160.8 ± 5.43a	47.9 ± 0.73b
	USL	32.98 ± 0.31a	3.41 ± 0.02a	70.24 ± 0.42e	15.0 ± 0.42a	0.25 ± 0.02a	128.23 ± 0.62b	0.78 ± 0.02a	45.68 ± 0.24a	2.4 ± 0.27a	150.9 ± 4.34ab	48.1 ± 0.74b
W/R/FR	ISL	22.53 ± 0.12d	2.48 ± 0.02d	79.51 ± 0.53bc	13.4 ± 0.43d	0.22 ± 0.02a	114.78 ± 0.82d	0.42 ± 0.01e	45.32 ± 0.51a	1.4 ± 0.07c	161.5 ± 6.51a	43.7 ± 1.11c
	USL	25.07 ± 0.31c	2.83 ± 0.03c	77.42 ± 0.12cd	14.1 ± 0.12bc	0.23 ± 0.02a	115.03 ± 0.71d	0.50 ± 0.02d	45.17 ± 0.34a	1.6 ± 0.10bc	129.4 ± 4.34c	43.8 ± 1.08c
W/B	ISL	25.28 ± 0.32c	2.80 ± 0.03c	75.28 ± 0.68d	14.5 ± 0.28b	0.25 ± 0.03a	128.14 ± 0.93b	0.54 ± 0.01cd	45.61 ± 0.42a	2.4 ± 0.33a	140.9 ± 2.42b	49.6 ± 0.52ab
	USL	33.01 ± 0.31a	3.41 ± 0.01a	71.01 ± 0.23e	15.2 ± 0.34a	0.27 ± 0.01a	133.32 ± 0.83a	0.78 ± 0.03a	45.51 ± 0.63a	2.5 ± 0.26a	144.4 ± 2.33b	50.5 ± 0.93a
Quality		^*^	^*^	^*^	N.S.	N.S.	^*^	^*^	^*^	^*^	^*^	^*^
Orientation		^*^	^*^	^*^	^*^	N.S.	N.S.	^*^	N.S.	^*^	N.S.	^*^
Quality × Orientation		N.S.	N.S.	^*^	^*^	N.S.	^*^	^*^	N.S.	^*^	^*^	^*^

*Supplemental lighting from the underneath canopy (USL) or from the inner canopy (ISL) was applied to plants from the time of transplantation while a no supplemental lighting condition was considered to be the control (CK). Means ± SE (n = 8) with different letters within each row indicating significant difference by Duncan's multiple range test at P < 0.05*.

* *, significant by two-way ANOVA at P < 0.05; N.S., non-significant*.

### Correlation analysis of growth, photosynthesis and stomatal parameters

The aperture width/length, stomatal pore area and stomatal index, *F*_v_/*F*_m_, ΦPSII, starch content, stem dry weight, specific leaf area, flower number, and health index were highly significantly correlated to photosynthetic performance and growth development of tomato plants (*P* < 0.05; Table 2). Furthermore, two-way ANOVA analysis showed that there were highly significant effects of light quality, light orientation, and interaction factors between the quality × orientation on the growth, stomatal and gas exchange parameters (Tables 1, 3). However, there were no significant quality effects on the stem diameter, leaf area (Table 1), *F*′_v_/$F_{\text{m}}^{\prime }$, stomatal density, and aperture length (Table 3). The orientation had no effects on the leaf area, specific leaf area, chlorophyll content, soluble sugar content (Table 1), *F*_v_/*F*_m_, stomatal index, aperture length, aperture width/aperture length (Table 3). There was also no significant quality × orientation effects on the shoot dry weight, root dry weight, leaf area (Table 1), *F*_v_/*F*_m_, stomatal index, and aperture length (Table 3).

**Table 2:** **Correlation analysis of selected stomatal parameters, photosynthetic characteristics and plant development of tomato plants under different treatments**.

**Parameter^a^**	**AW/AL**	**SPA**	**SI**	***P*_N_**	***G*_s_**	***F*_v_/*F*_m_**	**ΦPSII**	**SSC**	**SC**	**SH**	**SDW**	**RDW**	**SLA**	**FN**	**HI**
AW/WL	1														
SPA	0.966^*^^b^	1													
SI	0.884	0.930^*^	1												
*P*_N_	0.964^*^	0.957^*^	0.940^*^	1											
*G*_s_	0.960^*^	0.969^*^	0.942^*^	0.900	1										
*F*_v_/*F*_m_	0.882	0.889	0.848	0.946^*^	0.848	1									
ΦPSII	0.783	0.862	0.865	0.934^*^	0.875	0.816	1								
SSC	0.778	0.681	0.590	0.583	0.611	0.853	0.442	1							
SC	0.963^*^	0.944^*^	0.821	0.954^*^	0.905^*^	0.878	0.705	0.798	1						
SH	−0.752	−0.840	−0.897	−0.836	−0.856	−0.713	−0.901^*^	−0.364	−0.692	1					
SDW	0.847	0.904^*^	0.922^*^	0.901^*^	0.883	0.831	0.883	0.578	0.752	−0.903^*^	1				
RDW	0.905^*^	0.918^*^	0.932^*^	0.852	0.963^*^	0.891	0.935^*^	0.615	0.843	−0.867	0.865	1			
SLA	0.919^*^	0.950^*^	0.859	0.908^*^	0.926^*^	0.816	0.729	0.634	0.944^*^	−0.777	0.818	0.834	1		
FN	0.915^*^	0.965^*^	0.902^*^	0.958^*^	0.929^*^	0.815	0.753	0.616	0.936^*^	−0.771	0.809	0.846	0.967^*^	1	
HI	0.916^*^	0.921^*^	0.900^*^	0.936^*^	0.885	0.774	0.899	0.461	0.757	−0.965^*^	0.965^*^	0.860	0.853	0.828	1

a *Aperture wide/aperture length (AW/AL), stomatal pore area (SPA), stomatal index (SI), net photosynthesis rate (P_N_), stomatal conductance (G_s_), maximum quantum yield of the PSII primary photochemistry (F_v_/F_m_), quantum yield of PSII electron transport (ΦPSII), soluble sugar content (SSC), starch content (SC), stem height (SH), stem dry weight (SDW), root dry weight (RDW), specific leaf area (SLA), flower number (FN), health index (HI)*.

b, * *, significant by t-test at P < 0.05*.

**Table 3:** **Two-way ANOVA analysis of the effects of supplemental light quality, light orientation, and quality × orientation interaction on photosynthetic characteristics and stomatal parameters of tomato plants under different treatment conditions**.

**Parameter ^a^**	***P*_N_**	***G*_s_**	***T*_r_**	***C*_i_**	***P*_Nmax_**	**AQY**	***A*_max_**	**CE**	***F*_v_/*F*_m_**	***F*′_v_/*F*′_m_**	**ΦPSII**	***q*N**	**SD**	**SI**	**AL**	**AW**	**AW/AL**	**SPA**
Quality	^*^^b^	^*^	^*^	^*^	^*^	^*^	^*^	^*^	^*^	N.S.	^*^	^*^	N.S.	^*^	N.S.	^*^	^*^	^*^
Orientation	^*^	^*^	^*^	^*^	^*^	^*^	^*^	^*^	N.S.	^*^	^*^	^*^	^*^	N.S.	N.S.	^*^	N.S.	^*^
Quality × Orientation	^*^	^*^	^*^	^*^	^*^	^*^	^*^	^*^	N.S.	^*^	^*^	^*^	^*^	N.S.	N.S.	^*^	^*^	^*^

a *Net photosynthesis rate (P_N_), stomatal conductance (G_s_), transpiration rate (T_r_), intercellular CO_2_ concentration (C_i_), light-saturated maximum photosynthetic rate (P_Nmax_), apparent quantum yield (AQY), CO_2_-saturated maximum photosynthetic rate (A_max_), carboxylation efficiency (CE), maximum quantum yield of the PSII primary photochemistry (F_v_/F_m_), efficiency of excitation energy capture by open PSII reaction centers (F′v/F′m), quantum yield of PSII electron transport (ΦPSII), non-photochemical quenching (qN), stomatal density (SD), stomatal index (SI), aperture length (AL), aperture width (AW), aperture wide/aperture length (AW/AL), stomatal pore area (SPA)*.

b, * *, significant by two-way ANOVA at P < 0.05; N.S., non-significant*.

## Discussion

Plant morphogenesis and development are controlled by genetics as well as environmental factors such as light quality, intensity, and orientation (Pearcy, 1988, 1990; Aldesuquy et al., 2000; Naumburg and Ellsworth, 2002; Hogewoning et al., 2010a,b; Wahidin et al., 2013). Low-light conditions in intensive greenhouse crop cultivation will reduce the plant growth and restrict the productive capacity (Demers et al., 1998; Frantz et al., 2000; Demers and Gosselin, 2002; Steinger et al., 2003; Shimazaki et al., 2007). Our results demonstrated that supplemental lighting treatment significantly improved the photosynthetic performance and stomatal regulations, thus improving the biomass production of tomato plants.

Plant photosynthesis is extremely sensitive to supra-optimal light conditions. Low light stress can damage the photosynthetic apparatus (Naumburg and Ellsworth, 2002), degrade the photosynthetic pigments (Aldesuquy et al., 2000), and suppress the carbon assimilation (Nawrocki et al., 2015). In our study, supplemental lighting treatment was found to improve the photosynthesis ability of leaves (Figures 2–4), which was in accordance with the previous work done to other species (Hovi et al., 2004; Hovi and Tahvonen, 2008; Massa et al., 2008). Chlorophyll captures light and soaks up the energy from it. The chlorophyll content closely related to the photosynthesis ability of leaf (Tewolde et al., 2016), and lack of the light-harvesting complex will affect chloroplasts structure and decrease chlorophyll content (Kovács et al., 2006). Powles (1984) had found that when plant suffered from photoinhibition induced by visible light, the photosynthetic apparatus was injured and chlorophyll content decreased dramatically, while Sokawa and Hase (1967) declared that in low light condition, enhanced light illumination would trigger chlorophyll formation and accompanied with increased light harvesting and modified photoreceptor. In this study, the chlorophyll content was enhanced in plants treated with supplemental lighting (SPAD, Table 1), which indicated the improvement of photosynthetic apparatus integrity and light harvesting efficiency. However, there was no significant difference in the chlorophyll content among the supplemental lighting treatments, indicating that the variation in the increased *P*_N_ among the supplemental lighting treatments was probably due to variations in the CO_2_ supply (the quantity that entered leaf through stomatal aperture, not the ambient CO_2_ concentration) and/or assimilation efficiency. We observed significantly higher *P*_N_ and *G*_s_ (Figures 2A,B) in the W/R/B and W/B treatment conditions, indicating that plants treated with these types of polychromatic supplemental lighting had performed better CO_2_ utilization efficiency. Considering the relative spectral distribution, there are larger proportions of blue light in W/R/B and W/B. Tough pure blue light has negative effects on photosynthesis, especially on tree species (McCree, 1972; Sarala et al., 2009; Pallozzi et al., 2013), adding blue light to the other spectrum could stimulate photosynthesis in wheat (Goins et al., 1997) and tomato (Arena et al., 2016). Sharkey and Raschke (1981) found that blue light could induce stomatal opening, thus increasing the stomatal pore area (**Figure 7D**) allowing for a higher availability of CO_2_ in the mesophyll. The data of *P*_Nmax_, *A*_max_, AQY, and CE (Figures 3C,F) are also significantly higher in W/R/B and W/B. *P*_Nmax_ and *A*_max_ are related to the activities of photosynthetic electron transport and phosphorylation. AQY represents CO_2_ assimilation or oxygen release when one photon is absorbed by the plant, and CE represents the carboxylation efficiency (Farquhar et al., 1980; Reng et al., 2003). These improved photosynthetic parameters confirmed the hypothesis that the enhanced blue light fraction in polychromatic illumination could promote photosynthetic electron transport activity and enhance the CO_2_ assimilation efficiency. This result was in consistent with the findings of Hogewoning et al. (2010b), who determined that the photosynthetic capacity of cucumber leaves increased as the blue light fraction increased. Chlorophyll fluorescence parameter variations provided the further explanation of optimized photosynthetic regulation under SL treatment. We observed significantly higher ΦPSII (Figure 4C) and NPQ (Figure 4D) under the W/R/B and W/B treatments. ΦPSII represents the electron supply for photosynthesis, highly correlated to *P*_N_ (Table 2), while NPQ suggests excessive energy dissipation ability, a most common form of photo protector against stress (Maxwell and Johnson, 2000). Thus, W/R/B and W/B improved the actual quantum yield of PSII electron transport and relieved light insufficiency stress in tomato leaves.

On the other side, compared with the control, plants under the treatments of supplemental lighting from the inner canopy generally presented with increased *P*_N_, *G*_s_, *C*_i_, and *T*_r_ (Figures 2A–D), which indicated that, in addition to the enhancement of chlorophyll content (Table 1), the increase in *P*_N_ was mostly caused by improved stomatal conductance, which provide sufficient CO_2_ for photosynthesis (Farquhar and Sharkey, 1982). This result was in accordance with the research on cucumber (Hao and Papadopoulos, 1999), which demonstrates that after treatment of supplemental lighting, the *P*_N_ was increased with *G*_s_ and expanded stomatal aperture. However, in the treatments of supplemental lighting from the underneath canopy, accompanied with an increase in the *P*_N_, *G*_s_, and *T*_r_ of tomato plants, *C*_i_ was unaffected compared with the control. Combining the increased in *P*_Nmax_, *A*_max_, AQY, and CE (Figures 3C–F), these results suggest that in addition to the influence from chlorophyll content, the increase in the *P*_N_ by supplemental lighting from the underneath canopy was mostly related to the highly improved CO_2_ assimilation efficiency, rather than to the simply enhanced CO_2_ supply. Studies on the effects of abaxial lighting treatment on plant photosynthesis in *Paspalum dilatatum* (Soares et al., 2008) and *Helianthus annuus* (Wang et al., 2008) also showed photosynthesis improvements closely related to CE. Given that *T*_r_ is similar to that in treatments of supplemental lighting from the inner canopy, the CO_2_ assimilation efficiency should be the determining factor of *P*_N_ variation between the treatments of supplemental lighting from the underneath and inner canopy conditions. *F*_v_/*F*_m_ (Figure 4A) was not statistically changed, reconfirming that the variation of increased *P*_N_ among supplemental lighting treatments was due to variation in CO_2_ utilization and independent of light-harvesting. However, the increased *F*′_v_/$F_{\text{m}}^{\prime }$, ΦPSII, and NPQ (Figures 4B–D) suggested that supplemental lighting from the underneath canopy improved the quantum yield of PSII electron transport and the excessive energy dissipation ability of tomato leaves. The increased ΦPSII meant that the majority of the photons absorbed by PSII and used in photochemistry were promoted to increase the level of the photorespiration rate. Therefore, the supplemental lighting from the underneath canopy could promote quantum yields of both PSII electron transport and carboxylation rates of tomato plants, leading to an increase in the photosynthetic efficiency, which is in accordance with the observed photosynthesis improvements by the application of abaxial lighting treatment on sunflower plants (Wang et al., 2008). In this study, the supplemental lighting from the inner canopy did not affect *F*′_v_/$F_{\text{m}}^{\prime }$ (Figure 4B), and only partly increased ΦPSII and NPQ (in W/R/B and W/B, Figures 4C,D). Data of the above parameters were significantly lower than those in the treatments of supplemental lighting from the underneath canopy. This results reconfirmed the lower carboxylation efficiency in plants treated with supplemental lighting from inner canopy, and this induced a relatively lower *P*_N_, compared with the other kind of light orientation treatment (Figure 2).

Stomatal morphogenesis and behavior are controlled by genetic as well as environmental factors, such as in light (Meckel et al., 2007; Mott et al., 2008; O'Carrigan et al., 2014). In this study, stomatal density was not affected by the light quality of supplemental lighting, and the stomatal index was not affected by the supplemental lighting orientation and quality × orientation (Figure 6; Table 3). However, the stomatal form and aperture size was significantly affected by the supplemental lighting conditions (Figures 5, 7). The aperture width was significantly increased in supplemental lighting treatment, accompanied by an increased aperture width/length ratio and stomatal pore area (Figure 7), suggesting that supplemental lighting could remit stomatal closure to promote enter-cell CO_2_ supply other than enhancing the stomatal number, which was in accordance with previous research on the cowpea (Schoch et al., 1980) and other tomato species (Gay and Hurd, 1975; Lee et al., 2007). The stomatal closure, usually induced by environmental stress, prevents CO_2_ from entering the mesophyll cells (Mott et al., 2008; Araújo et al., 2011) and decreases the internal CO_2_ concentration (Lake et al., 2001). Additionally, the stomatal morphology and density are correlated with leaf photosynthesis and plant development (Table 2). The aperture width/length and stomatal pore area are highly positively correlated to the *P*_N_, *G*_s_, specific leaf area, flower number, and health index, but are negatively linked to the stem height (Table 2). This was in accordance with the work of O'Carrigan et al. (2014), who found that a decrease in the aperture area could reduce the *P*_N_ of tomato leave and induce excessive plant growth and a decrease in the flower number. These results indicated that the enlargement of the aperture could increase the CO_2_ supply and that stomatal morphology should be an important determinant of photosynthesis and growth of greenhouse cultivated tomato.

Enhanced leaf photosynthesis capacity and optimized stomatal regulation can enhance plant development (Hovi et al., 2004; Hovi and Tahvonen, 2008; Pettersen et al., 2010; O'Carrigan et al., 2014). In this study, tomato morphological features were notably influenced by the application of supplemental lighting (Table 1), reconfirming that plant morphology could be improved by increasing the light intensity (Seibert et al., 1975; Marschner and Cakmak, 1989) and also demonstrating the feasibility of cultivating tomato intensively through the application of supplemental lighting to the lower canopy. The remarkably improved plant profile (dry weight of both stems and roots, specific leaf area, health index) and reproductive speed (flower number) in W/R/B and W/B (Table 1), indicating a decreased unit cultivation period and an increased annual production time, which potentially improves the benefit return of this technique. These results reconfirmed that the enlarging blue light fraction in polychromatic illumination has better performance. Meanwhile, the indoleacetic acid (IAA) oxidase activity can be promoted by enhancing blue light proportion in illumination, which decreases the IAA level, consequently preventing excessive growth and guaranteeing reproductive development in various species, such as broad bean (Assmann et al., 1985), pepper (Brown et al., 1995), and lettuce (Li and Kubota, 2009). Additionally, under red light conditions, adding blue light irradiation could trigger epidermal cell elongation of the abaxial side and inhibit leaf epinasty in the geranium (Fukuda et al., 2008), results in more direct irradiation interception. Although R/B consisted of blue light, the green light spectrum was added to the W/R/B and W/B, and the addition of green light could enhance the photochemical content (Li and Kubota, 2009) and drive leaf photosynthesis more efficiently than red light (Terashima et al., 2009). A large proportional increase in the far-red light could significantly limit the biomass accumulation (Wang et al., 2009), which explained the inhibition of tomato plant growth in W/R/FR condition compared with other supplemental lighting treatment conditions.

## Conclusion

Supplemental lighting with polychromatic light applied from either inner canopy or underneath canopy effectively increased tomato photosynthetic efficiency, reduced stomatal closure and improved plant development. W/R/B and W/B from underneath canopy promoted plants with higher health index and faster development, and presented better performance. CO_2_ utilization efficiency determined the variation of photosynthetic performance among the supplemental lighting treatments. An enhanced blue light fraction in W/R/B and W/B could better stimulate stomatal opening and promote photosynthetic electron transport activity, thus better improving photosynthetic rate. The mechanisms of photosynthesis improvement differed for the two light orientation treatments. The supplemental lighting from the inner canopy improved the photosynthesis of tomato plants by increasing the stomatal conductance to enhance the CO_2_ supply for leaf, thereby promoting photosynthetic electron transport activity. The supplemental lighting from the underneath canopy improved photosynthesis by enhancing the CO_2_ supply as well as increasing the CO_2_ assimilation efficiency and excessive energy dissipation, of which the enhancement contributed to a higher photosynthetic rate compared with the treatment of supplemental lighting from the inner canopy. Stomatal morphology was highly positively associated with leaf photosynthesis and plant development, and is therefore believed to be an important determinant for photosynthesis and growth of greenhouse cultivated tomato.

## Author contributions

YS and CJ contribute equally to this manuscript. YS, CJ, and LG conceived and designed the experiments. YS performed the experiments. YS and CJ analyzed the data and prepared the manuscript.

## Funding

This work was supported by Xinjiang Major Project: Research and Demonstration of Critical Technology for Efficient and Sustainable Development of Xinjiang Agricultural Industrial Facilities (No. 201130104).

### Conflict of interest statement

The authors declare that the research was conducted in the absence of any commercial or financial relationships that could be construed as a potential conflict of interest.
